# Carbon Dots for Intracellular pH Sensing with Fluorescence Lifetime Imaging Microscopy

**DOI:** 10.3390/nano10040604

**Published:** 2020-03-25

**Authors:** Maojia Huang, Xinyue Liang, Zixiao Zhang, Jing Wang, Yiyan Fei, Jiong Ma, Songnan Qu, Lan Mi

**Affiliations:** 1 Department of Optical Science and Engineering, Shanghai Engineering Research Center of Ultra-precision Optical Manufacturing, Green Photoelectron Platform, Fudan University, Shanghai 200433, China; 17210720002@fudan.edu.cn (M.H.); 18210720003@fudan.edu.cn (X.L.); 16307130097@fudan.edu.cn (Z.Z.); fyy@fudan.edu.cn (Y.F.); 2 State Key Laboratory of High Field Laser Physics, Shanghai Institute of Optics and Fine Mechanics, Chinese Academy of Sciences, Shanghai 201800, China; wangjing@siom.ac.cn; 3 Institute of Biomedical Engineering and Technology, Academy for Engineer and Technology, Fudan University, Shanghai 200433, China; 4 The Multiscale Research Institute of Complex Systems (MRICS), School of Life Sciences, Fudan University, Shanghai 200433, China; 5 Joint Key Laboratory of the Ministry of Education, Institute of Applied Physics and Materials Engineering, University of Macau, Avenida da Universidade, Taipa, Macau 999078, China

**Keywords:** carbon dots, fluorescence lifetime imaging microscopy, pH sensor, intracellular sensing

## Abstract

The monitoring of intracellular pH is of great importance for understanding intracellular trafficking and functions. It has various limitations for biosensing based on the fluorescence intensity or spectra study. In this research, pH-sensitive carbon dots (CDs) were employed for intracellular pH sensing with fluorescence lifetime imaging microscopy (FLIM) for the first time. FLIM is a highly sensitive method that is used to detect a microenvironment and it can overcome the limitations of biosensing methods based on fluorescence intensity. The different groups on the CDs surfaces changing with pH environments led to different fluorescence lifetime values. The CDs aqueous solution had a gradual change from 1.6 ns to 3.7 ns in the fluorescence lifetime with a pH range of 2.6–8.6. Similar fluorescence lifetime changes were found in pH buffer-treated living cells. The detection of lysosomes, cytoplasm, and nuclei in living cells was achieved by measuring the fluorescence lifetime of CDs. In particular, a phasor FLIM analysis was used to improve the pH imaging. Moreover, the effects of the coenzymes, amino acids, and proteins on the fluorescence lifetime of CDs were examined in order to mimic the complex microenvironment inside the cells.

## 1. Introduction

Intracellular pH is of great importance for maintaining a normal cell activity [1], which plays a crucial role in biological functions such as cell proliferation [2], apoptosis [3], ion transport [4], endocytosis [5], and tumor growth [6]. Abnormal pH can lead to a variety of diseases such as cancer [7] and Alzheimer’s disease [8]. The real-time monitoring of the intracellular pH may aid understanding of the pathogenesis of these diseases and aid with treatment. Therefore, monitoring intracellular pH in real time is of great significance. Among the traditional approaches for measuring intracellular pH, fluorescence technologies show important advantages due to non-invasiveness, high sensitivity and low cost [9].Organic dyes [10,11], fluorescent proteins [12], and fluorescent nanomaterials [13] including quantum dots (QDs) have been applied to measure intracellular pH. However, there are various challenges for them. Organic dyes usually a have high susceptibility to photobleaching and relatively narrow absorption [14]. Fluorescent proteins often show poor permeability [15]. QDs are the most familiar fluorescent nanomaterials with high brightness and good photostability, but most of the QDs containing heavy metal components such as cadmium, mercury or lead, are highly toxic [16]. Polyethylenimine-coated upconversion of nanoprobes was reported with large particle size and not conductive to cell imaging though its ratiometric sensing in solutions can be achieved [17].

Carbon dots (CDs) have received extensive attention in recent years due to their stable optical properties [18], good biocompatibility [19], and low toxicity [20]. CDs have shown great potential in the study of bioimaging [21,22], photodynamic therapy [23], biosensing [24,25], drug carriers [26,27], and disease treatment [28]. In recent years, various CD-based nanoprobe strategies for intracellular pH sensing have emerged. Most of the strategies can be divided into two types: One type is based on the increase or decrease of fluorescence intensity [21,29,30]. Wu et al. reported nitrogen-doped graphene quantum dots as a quantitative pH sensor, and the increase of the fluorescence intensity was observed with the increase of the pH values [31]. Chandra et al. synthesized pH-sensitive CDs, the fluorescence intensity of which increased as the pH was changed from 7.4 to 5.0 [21]. Recently, Wang et al. reported that the fluorescence intensity of CDs depends on the pH value in the range from 6.0 to 10.0 and showed excellent stability against the influence of metal ions and amino acids [32]. Yao et al. reported a CDs@UiO-66(OH)_2_ composite that can be effective for the detection of temperature, pH, and Fe^3+^ [33]. Zhang et al. found a linear relationship between the fluorescent intensity and pH (1.0–3.0) and the reversible pH-response of the fluorescence behavior of N,S-CDs between pH 1.0 and 13.0 [34]. However, fluorescence intensity-based measurements suffer effects such as excitation laser stability, concentration of fluorescent probes, scattering and absorption of biological complex systems, and fluorescence quenching. Therefore, in the first type of strategies, it is hard to obtain the pH values in the cells quantitatively. The other type of strategies involve using probes with two separate wavelength emissions whose ratio of two emission intensities corresponds to ratiometric pH values [14,35,36], or with a red or blue shift [37,38,39,40]. For example, Yuan et al. observed the absorption redshift from 292 to 316 nm and 525 to 560 nm when pH was increased from 1 to 11 and 12 to 14, respectively [40]. Zhang et al. reported that the fluorescence intensity decayed from pH = 2 to 12. However, the peak location was almost unchanged from pH = 2 to 6, and the peak location from pH 7 to 12 redshift from 458 to 491 nm under excitation at 350 nm [39]. This type of strategy can overcome some of the aforementioned impacts, but the emission properties may be altered in a complex biological system such as in certain compartments of cells [41]. A report of N-doped CDs in 2015 by Zheng et al. demonstrated that the pH value influenced the absorbance, the emission peaks, as well as the fluorescence lifetime [42]. However, the excitation and emission wavelengths in this report were mainly in UV range, which would limit the application in biological research.

**Scheme 1:**
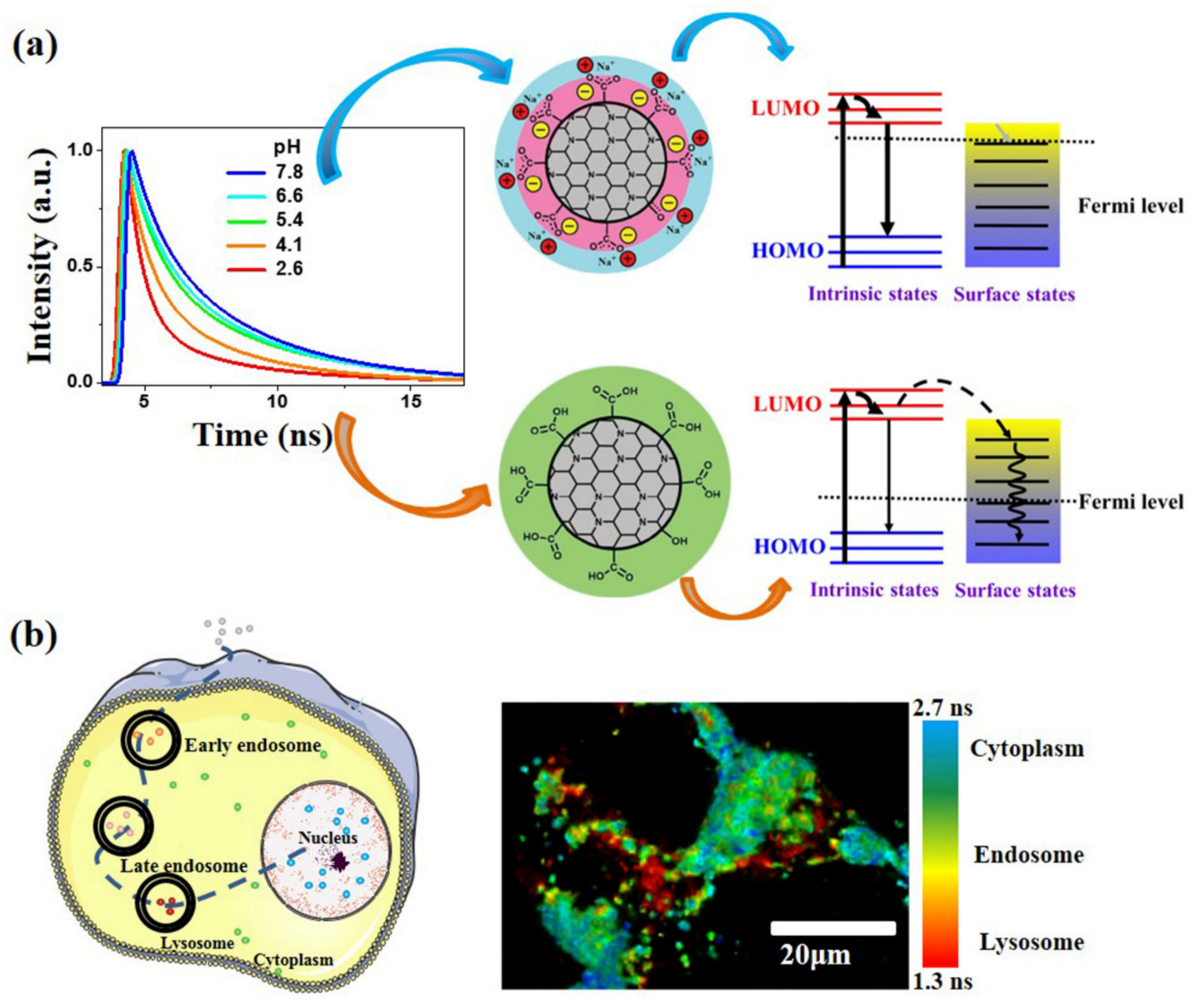
(**a**) FLIM measurement of CDs aqueous solutions under different pH conditions showing different fluorescence lifetimes, and a possible mechanism schematic diagram for CDs under different pH conditions. (**b**) Schematic illustration of the intracellular delivery of carbon dots and the FLIM 3D remodeling image of CDs in cells. The CDs had different fluorescence lifetimes in different regions such as cytoplasm, endosomes, and lysosomes, showing different colors. Scale bar: 20 μm.

Fluorescence lifetime imaging microscopy (FLIM) is a highly sensitive method used to detect the microenvironment of fluorescent probes. Furthermore, it is independent of the concentration of fluorescent probe or excitation power. There are various types of materials reported as tools for pH sensing, including dyes [43], fluorescent proteins [44,45], quantum dots [41]. Considering the low cytotoxicity and high photostability of CDs, we propose pH-sensitive CDs for intracellular pH sensing by FLIM (Scheme 1). As we reported previously, the CDs were prepared via a solvothermal route from citric acid and urea, with abundant carboxyl, hydroxyl, and amide groups on the surface [46]. The carboxyl and phenolic hydroxyl groups were neutralized when adding an alkali solution and the surface functionalized metal cations could be further taken off in acidic solution [46]. The different groups on the CDs surface that changed with the pH environments led to different fluorescence lifetime values. The cytotoxicity of CDs is quite low and negligible (see Supplementary Figure S1). In addition, our previous toxicity evaluation work for CDs found no to low toxic effect on a wide range of cell lines [47] and mice [48]. Thus, our low-toxic and pH-sensitive CDs can be safely employed for intracellular pH sensing, instead of cadmium-containing quantum dots [41].

In this research, we demonstrated that CDs had a gradual change in fluorescence lifetime in the pH range of 2.6–8.6. Three-dimensional (3D) FLIM imaging was achieved for CD-labeled living cells. CDs in lysosomes, cytoplasm, and nucleus were further studied. A phasor FLIM analysis was used to improve the pH imaging. By studying the effects of the different components of cells (such as coenzymes, amino acids, and proteins) on the CDs fluorescence, the changes of the CDs fluorescence lifetime in the complex microenvironment inside cells were partially explained.

## 2. Materials and Methods

### 2.1. Synthesis and Solution Preparation of CDs

The CDs were synthesized according to our previous report [46]. Briefly, the CDs were synthesized using the solvothermal method with urea and citric acid as the carbon source in dimethylformamide (DMF) at 160 °C for 6 h. After cooling to room temperature, the obtained solution was mixed with alkali (NaOH or KOH) aqueous solution to obtain the precipitate. After lyophilization, the solution was dissolved in dilute HCl aqueous and then it was centrifuged and lyophilized to obtain the powder of the CDs. The CDs were dissolved in dimethyl sulfoxide (DMSO) solution at a concentration of 4 mg mL^−1^. The CDs solution was diluted with deionized water (DH_2_O) at a final concentration of 0.05 mg mL^−1^ whose absorbance and fluorescence spectra were shown in Supplementary Figure S2.

In order to measure the CDs solutions with different pH values, the CDs solutions were added 1M HCl or NaOH aqueous solution dropwise, to obtain the CDs solutions with a variety of pH values ranging from 2.0 to 8.6.

### 2.2. Buffer Solutions for Cells with Different pH Values

The reagent materials involved in the preparation of the pH buffer include nigericin, NaOH, MgSO_4_, KOH, KCL, K_2_HPO_4_, and CaCl_2_. The pH buffer was prepared by mixing appropriate proportions of the solutions of 135 mM KCl, 2 mM K_2_HPO_4_, 20 mM HEPES, 1.2 mM CaCl_2_, 0.8 mM MgSO_4_, and 20 μM nigericin [49]. After that, the pH of the buffer solution was adjusted to between 3.0 and 9.0 with HCl and KOH.

### 2.3. Cell Culture and Processing

To observe the fluorescence of CDs in cells, human cervical cancer (HeLa) cells were cultured in Petri dishes. HeLa cells were grown in a DMEM medium (Dulbecco’s modified Eagle’s medium, Gibco, New York, USA) supplemented with 10% (*v/v*) fetal calf serum (FBS, Gibco, New York, USA) at 37 °C in a humidified incubator with 5% CO_2_ until the growth density of the viable cells reached 80% in the culture dish. Then HeLa cells were incubated with CDs (0.2 mg mL^−1^ in DMEM) for 2 h at 37 °C. The cells were washed three times with PBS before observation.

To compare the CDs with a pH commercial probe, HeLa cells were incubated with Oregon Green^TM^ 488 Carboxylic Acid, Succinimidyl Ester, 5-isomer (Oregon Green, Thermo Fisher Scientific, Waltham, MA, USA). When the HeLa cells were seeded in dishes and they reached a density of 80%, Oregon Green (10 μg mL^−1^ in DMEM) was added to the dishes at 37 °C for 1 h and the cells were washed three times with PBS before observation.

For the above methods of treating cells, if it was necessary to adjust the intracellular pH environment, the above-mentioned treated cell dishes needed to have further pH buffer added for 10 min.

For lysosomal staining, the cells were incubated with the lysosomal specific probe, LysoTrackerTM Blue DND-22 (300 nM, ThermoFisher Scientific, Waltham, MA, USA) for 30 min.

When the cells with more permeability of the cell membranes and nuclear envelopes needed to be studied, the cells were treated with digitonin. The import buffer was prepared by mixing solutions of 20 mM HEPES, 110 nM KOAc, 5 mM NaOAc, 2 mM MgOAc, and 1 mM EGTA at a pH value of 7.3. After incubating the HeLa cells for 2 h with CDs (0.2 mg mL^−1^ in DMEM), the cells were washed three times with PBS and washed twice with import buffer. Then the cells were treated with digitonin (40 μg mL^−1^ in import buffer) for 2 min and washed with polyvinylpyrrolidon (PVP, 1.5% in import buffer).

For the cytotoxicity study, cells grown in 96-well plates (1 × 10^5^ cells per well), were incubated with 20 or 50 μg·mL^−1^ CDs dispersed in DMEM-H medium for 2 h in the dark. Then, the medium was removed and replaced by fresh culture medium, and the cells were incubated for 24 h. The cell viability assays were conducted by a modified MTT method using WST-8 (2-(2-methoxy-4-nitrophenyl)-3-(4-nitrophenyl)-5-(2,4-disulfophenyl)-2H tetrazolium, monosodium salt, Beyotime, Jiangsu, China). WST-8 produces a highly water soluble formazan upon cellular reduction and has found increasing applications for use in cell viability assays, while MTT suffers from the disadvantage of the insoluble formazan [50]. An amount of 10 μL of WST-8 was added in each well containing 100 μL culture medium. The cells were incubated at 37 °C with 5% CO_2_ for 2 h and the absorbance was measured at 450 nm using a microplate reader (Bio-Tek Synergy^TM^ HT, Bio-Tek Instruments Inc., Winooski, VT, USA). Cells incubated in DMEM-H medium without any treatment were used as the control group. Each experiment was conducted and measured independently for at least three times.

### 2.4. Fluorescence Spectra and Fluorescence Lifetime Imaging

The fluorescence spectra of the CDs solutions and the Oregon Green solutions under different pH values were measured by a fluorescence spectrometer F-2500 (Hitachi, Tokyo, Japan). All conditions were repeated at least five times. The reagents involved in the CDs solution measurement included bovine serum albumin (BSA, Sigma, St. Louis, MO, USA), histone (Sangon Biotech, Shanghai, China), glutamic acid and arginine (BBI Life Sciences), and phosphate buffered saline (PBS, Multicell, Wisent Inc., Quebec, Canada).

Fluorescence lifetime images were recorded with a time-correlated single photon counting (TCSPC) system (SPC-150, Becker & Hickl, Berlin, Germany) based on a confocal laser scanning microscope (Olympus, FV300/IX 71, Tokyo, Japan). The CDs or Oregon Green solutions at different pH values were excited by a 488 nm picosecond pulsed laser (BDL-488-SMN, Becker & Hickl, Berlin, Germany) with a repetition rate of 50 MHz using a 60×/N.A.1.2 water immersion objective lens. The fluorescence was collected by a photomultiplier tube (PMC-100-1, Becker & Hickl, Berlin, Germany) with a 496 nm longpass filter. For the solution experiments, all of the data were measured independently at least five times and then averaged. For the FLIM imaging of the cells, each condition was repeated at least three times, and 60–120 cells were collected for each condition for subsequent data analysis. No photobleaching effect was observed during the CDs-treated cells FLIM imaging within 15 min.

The cells labeled with Oregon Green were imaged by confocal fluorescence microscopy with a 488 nm continuous excitation laser. The fluorescence signal was collected by a photomultiplier tube with a 505–550 nm bandpass filter and a differential interference contrast (DIC) image of the transmission channel was simultaneously recorded for observation of the cell morphology.

### 2.5. FLIM Data Analysis

A multi-exponential fitting analysis was performed on the collected fluorescence lifetime data using SPCImage software (Becker & Hickl, Berlin, Germany). The average fluorescence lifetime was obtained from the weighted average of the fluorescence lifetimes of the different components, calculated using Equation (1):

(1) $$\tau_{m}=\overset{N}{\underset{i=1}{\sum }}a_{i}\tau_{i}/\overset{N}{\underset{i=1}{\sum }}a_{i}$$

where *τ_μ_* is the mean lifetime. *N* is the number of components. *τ_i_* and *a_i_* are the fluorescence lifetime and the proportion of the *i*-th component of the model, respectively. The multi-exponential fitting analysis of the fluorescence lifetime of the CDs showed that the triple-exponential fitting was better than single-exponential or double-exponential decay (Supplementary Figure S3 and Table S1). Thus, triple-exponential fitting for the fluorescence lifetime of the CDs was used in this research. The *τ_μ_* distribution curve of all pixels in each FLIM image can be obtained by the SPCImage software. The peak of the lifetime distribution curve was analysed for each FLIM image and more than five cell images were studied for each condition (Supplementary Figure S4).

### 2.6. Phasor FLIM Analysis

The analysis of Phasor FLIM was done by SimFCS software. Every pixel of the FLIM image was converted to one pixel of the phasor plot. The s and g coordinates within the phasor plot for the fluorescence decay curve *I*(*t*) were defined by the following expression [51]:

(2) $$g_{i,j}\left(\omega \right)=\frac{\int_{0}^{\infty }I_{i,j}\left(t\right)\cos\left(\omega t\right)dt}{\int_{0}^{\infty }I_{i,j}\left(t\right)dt},$$

(3) $$s_{i,j}\left(\omega \right)=\frac{\int_{0}^{\infty }I_{i,j}\left(t\right)\sin\left(\omega t\right)dt}{\int_{0}^{\infty }I_{i,j}\left(t\right)dt},$$

where *ω* = 2πφ and *f* is the laser repetition frequency. *i* and *j* define the pixels within the image.

In a system where the multi-component fluorescence lifetimes are mixed, the coordinates can be expressed as [51]:

(4) $$g_{i,j}\left(\omega \right)=\sum_{k}\frac{h_{k}}{1+{\left(\omega \tau_{k}\right)}^{2}},$$

(5) $$s_{i,j}\left(\omega \right)=\sum_{k}\frac{h_{k}\omega \tau_{k}}{1+{\left(\omega \tau_{k}\right)}^{2}},$$

where $h_{k}$ is the weighting factor of the component of a certain lifetime $\tau_{k}$.

The FLIM images were processed by simFCS software to obtain the scatter distribution of the corresponding phasor plots.

## 3. Results and Discussion

### 3.1. Fluorescence Characteristics of CDs in Different pH Solutions

**Figure 1:**
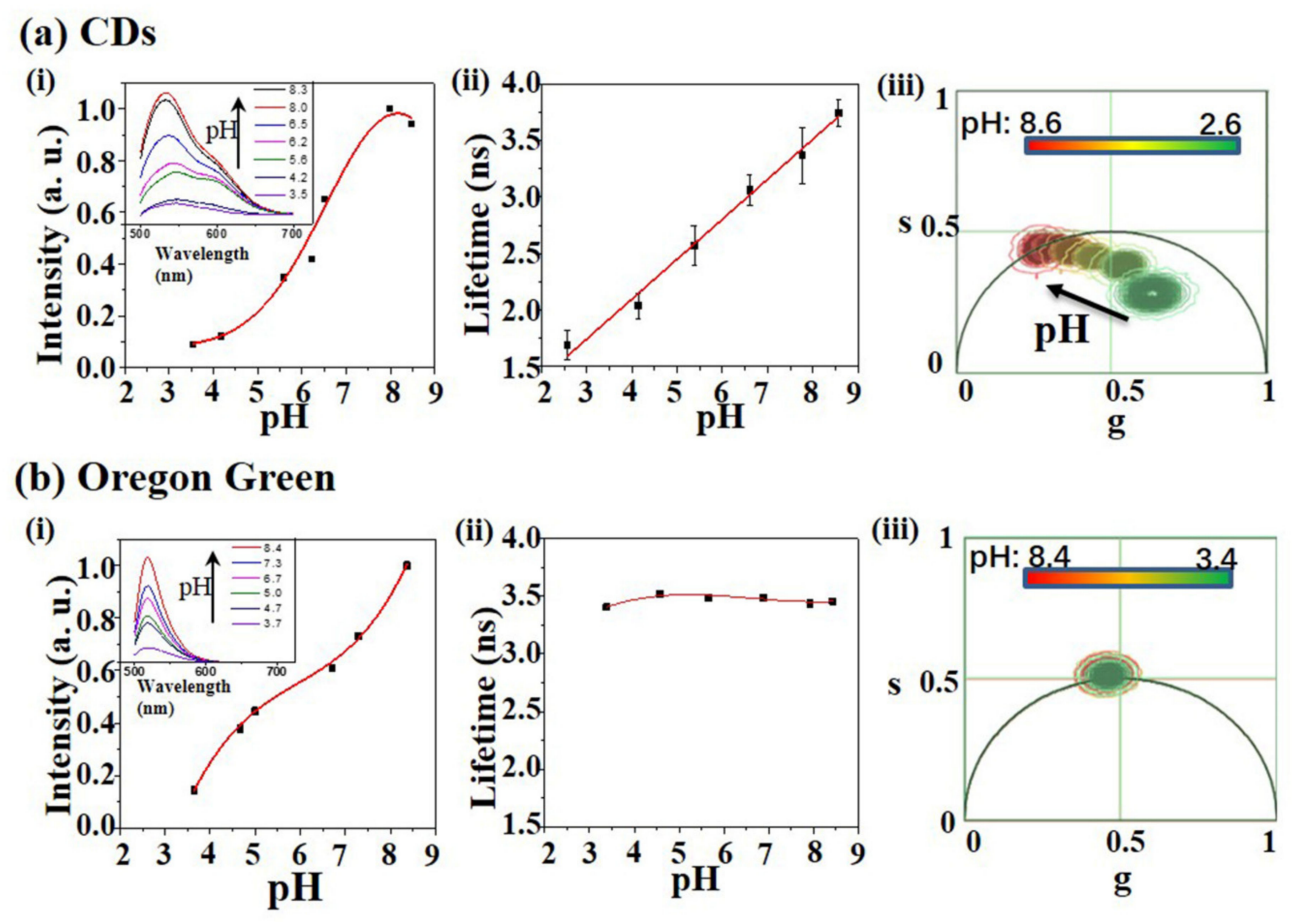
Comparison of the fluorescence characteristics of (**a**) CDs and (**b**) commercial pH sensor Oregon Green in different pH environments. (i) Fluorescence intensity changes with pH values (the error bar is very small in this case and the inset is the fluorescence spectra), (ii) fluorescence lifetime changes with pH values, and (iii) phasor analysis of FLIM images.

The fluorescence spectra of CDs solutions in different pH environments are shown in Figure 1a(i). The fluorescence intensity decreased in an acidic environment. When the pH value was lower than 3.5, the fluorescence spectrum was difficult to measure. The fluorescence intensity gradually increased with the increasing pH values, while the fluorescence intensity decreased a little when the pH exceeded 8.0. As reported by Yang et al., the fluorescence of CDs enhanced along with pH value increasing from 1.48 to 7.56, then slowed down when the pH reaches the values more than 8 [52]. This may be due to the deprotonation of surface groups of CDs at high pH values, and might form a new surface state [52]. As shown in Figure 1a(ii), the fluorescence lifetime of the CDs solutions with different pH values (pH = 2.6–8.6) increased linearly with the increase of the pH in the range of 1.6–3.7 ns. Based on the fitted linear line, the pH value of the solutions could be obtained from the fluorescence lifetime value of the CDs in the solutions. As shown in Figure 1a(iii), we performed a phasor analysis of the FLIM images of the CDs solutions. One of the major advantages of the phasor approach is that it is a fit-free method, and it has better universality than FLIM fitting analysis [51]. It can also be seen that as the pH increased, the phasor data gradually moved to the upper left, corresponding to a gradual increase in the fluorescence lifetime. A possible mechanism for the pH indicators of the CDs is illustrated in Scheme 1a. There are abundant carboxyl, hydroxyl, and amide groups on the surface of CDs. When in alkaline solution, carboxyl and phenolic hydroxyl groups are neutralized, while in acidic solution, the surface functionalized metal cations can be further taken off [46]. Surface metal cation functionalization could cause increased carboxylate ions on the inner surface of CDs in alkaline solution, leading to electron-rich property of the inner surface. The rich electrons could occupy the energy levels of the surface states and lift the Fermi level, leading to smaller self-absorption and enhancing the output emission of CDs in alkaline solution [46]. The mechanism of CDs fluorescence is still in debate, but there are a number of reports that agree that the protonation or deprotonation level of the surface functional groups of CDs may be different at variable pH environments, which could result in the change of fluorescence [31,53,54].

For comparison, a commercial pH probe, Oregon GreenTM 488 Carboxylic Acid, Succinimidyl Ester, 5-isomer (Oregon Green) was also studied in solutions with pH values in the range of 3.4–8.4. The fluorescence intensity of the Oregon Green increased with the increasing pH (Figure 1b(i)), and its fluorescence lifetime was essentially unchanged, about 3.4–3.5 ns in Figure 1b(ii). The corresponding phasor analysis map was also overlapped by multiple data (Figure 1b(iii)).

CDs can be used to determine pH based on not only the fluorescence intensity/spectrum but also the fluorescence lifetime, while Oregon Green can be used as a pH sensor based only on the fluorescence intensity measurements. Furthermore, various CDs were reported to be pH-sensitive by the fluorescence spectrum measurements in a wide pH range, but lacked intracellular quantification [32,34,55,56]. It is well known that the pH value is 4.0–5.5 in lysosomes, and around 7.4 in cytoplasma. Most of them cannot investigate the living cells in an appropriate pH range (from 4 to 8). Semiconducting QDs have been proven to determine intracellular pH by fluorescence lifetime [41,57]. CDs benefits over QDs for their easy synthesis, low cytotoxicity and good optical properties [58]. Therefore, for pH detection in complex biological environments, the present CDs have an advantage over the commercial pH probe Oregon Green and some other reported CDs.

### 3.2. CDs pH Response in Living Cells

Before observing the fluorescence of CDs in cells, the autofluorescence of control cells were compared with CDs-treated cells (Supplementary Figure S5). It was found that the autofluorescence intensity of cells was 8.5 times lower than the CDs fluorescence. The autofluorescence lifetime is around 0.98 ns. Thus, the autofluorescence signal does not affect the FLIM measurement of CDs-treated cells.

**Figure 2:**
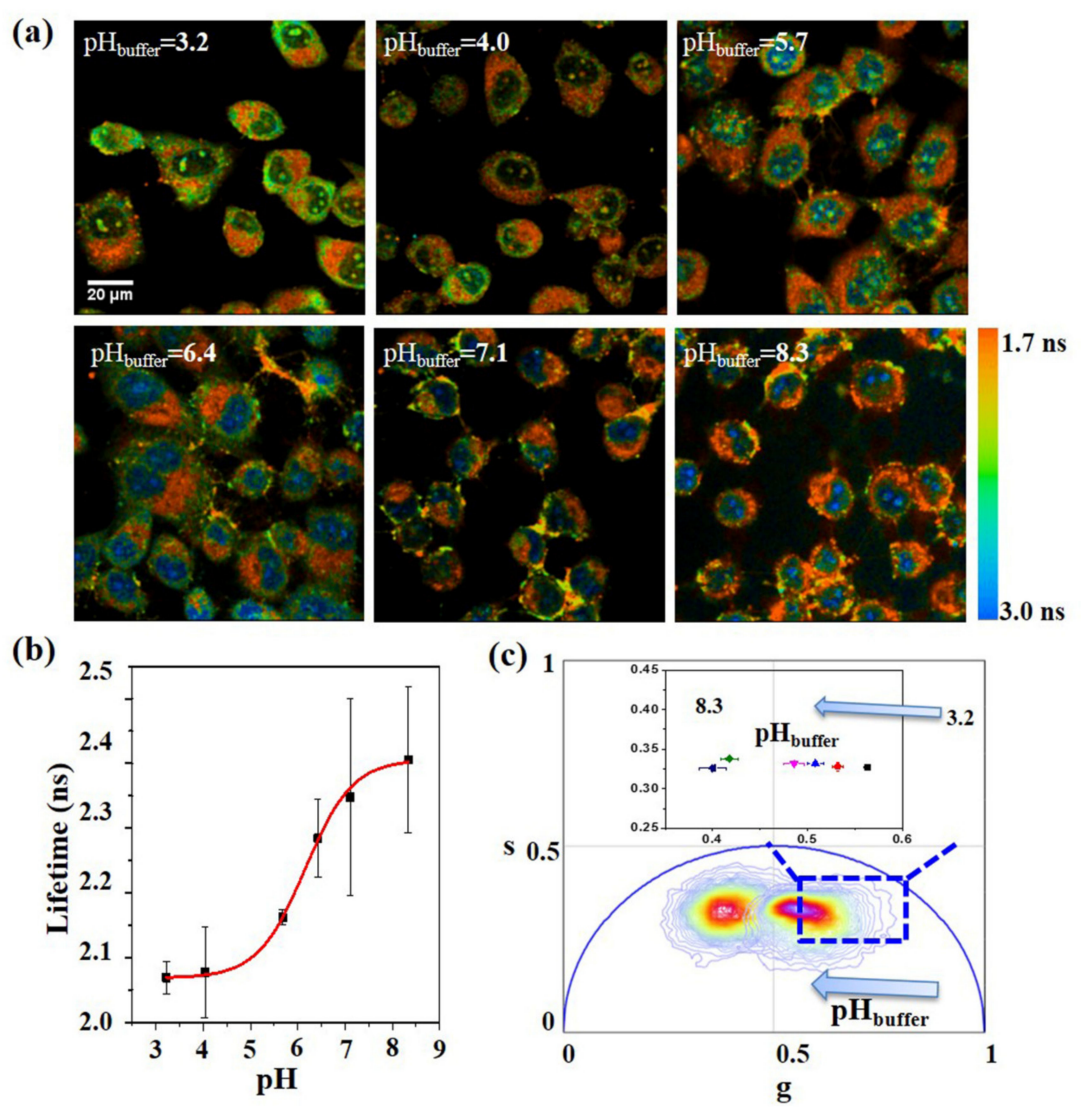
pH response of CDs in HeLa living cells. (**a**) Fluorescence lifetime images of cells labeled with CDs at different pH buffers, scale bar: 20 μm. (**b**) The relationship between the average fluorescence lifetime and the pH values of the FLIM images in (**a**). (**c**) A phasor diagram obtained using the phasor analysis of the FLIM images in (**a**), in which the enlarged part of the phasor diagram gives the maximum density of the corresponding phasor plot for each pH value.

The intracellular pH microenvironment could be changed by incubating cells with buffers of different pH values [41]. As shown in Figure 2a, when the HeLa cells were treated with different pH buffers, the intracellular pH was changed, but there were differences between the different regions of the cells. Therefore, different regions have different colors in the FLIM images of the CDs in the living cells. The overall fluorescence lifetime of the cells increased with the increasing buffer pH (Figure 2b). When the pH of the buffer was between 3.2 and 8.3, the average fluorescence lifetime of the CDs in the corresponding cells gradually increased from 2.1 ns to 2.4 ns. Compared with the linear increase in Figure 1a(ii), the curve is sigmoidal-like. This could be due to the intracellular pH being not the same as the pH value of buffer solutions, especially for the cells in alkaline and acidic environments. As reported in previous studies, the intracellular pH in mammalian cells is maintained within an optimal narrow range through the combined operation of transmembrane transporters and the intracellular buffering capacity [59,60]. The alkaline or acidic buffer solutions changed the intracellular pH, but the intracellular buffering and acid extrusion/loading systems trend to maintain intracellular pH neutral. Thus, it can be found that the changes of fluorescence lifetime become smaller in higher or lower pH values of buffers. Moreover, it should be noted that the fluorescence lifetime distribution of the intracellular CDs was quite different, and the difference between cells was large, so some error bars were fairly large in the statistics. A large error bar for the intensity ratio of intracellular pH sensing has also been observed for Boron-doped CDs in pH buffer treated HeLa cells [36].

In the phasor FLIM analysis (Figure 2c), the data of intracellular CDs shifted to the left as the pH increased. This trend is consistent with the trend of the phasor FLIM of the CDs solution in Figure 1a(iii). This demonstrates that the pH response of the CDs in the living cells was similar to that in the solutions.

In contrast, HeLa cells labelled with Oregon Green were also studied with confocal fluorescence imaging (Supplementary Figure S4), and the fluorescence intensity of the intracellular Oregon Green increased with the increasing buffer pH. However, it was difficult to distinguish the distribution of pH in different regions of the cell. Therefore, this method was not reliable and accurate in detecting the intracellular pH with fluorescence intensity measurements.

**Figure 3:**
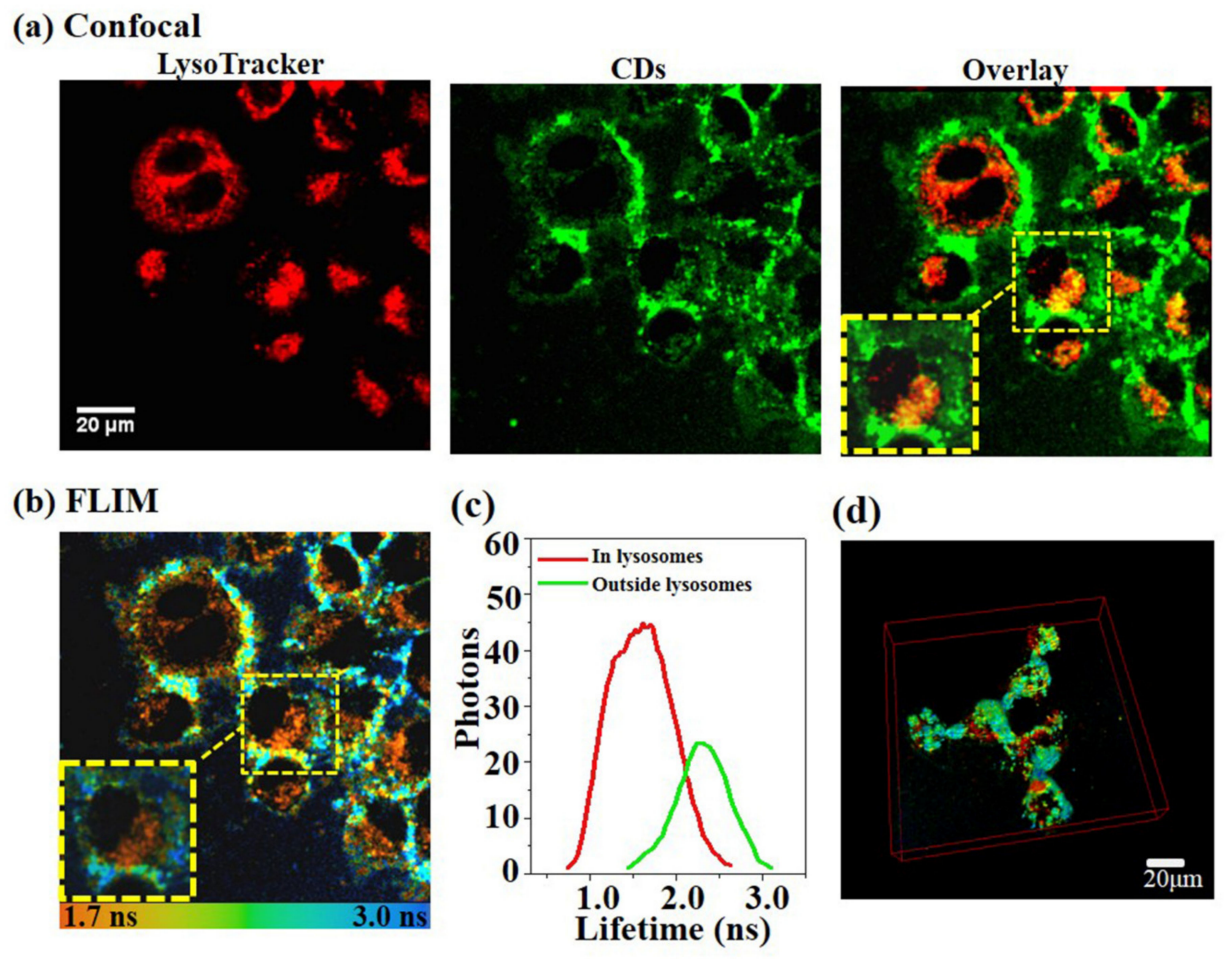
HeLa living cells incubated with CDs and a lysotracker. (**a**) Confocal microscopy of HeLa cells, left to right, lysosomes (red), CDs (green), and an overlay image, in which yellow indicates the co-localization of the CDs and lysosomes. (**b**) FLIM image of CDs in HeLa cells. (**c**) Statistical distribution of the fluorescence lifetimes of CDs inside lysosomes and outside lysosomes in HeLa cells. (**d**) Three-dimensional reconstruction of the FLIM images with 10 frames, 2.45 μm per step. The colors represent the average lifetime of the CDs, from red to blue, corresponding to 1.7–3.0 ns. Scale bar: 20 μm.

After changing the intracellular pH environment with a pH buffer, the permeability of the cell membrane was also affected [61]. To further study the pH response of CDs in the normal environment of living cells, HeLa cells were first incubated with CDs in PBS (Ph = 7.13) and then, the lysosomes of the cells were labeled with a lysotracker probe. The lysotracker probe was excited with a 405 nm laser and the CDs were excited by a 488 nm laser. As shown in Figure 3a, the lysotracker fluorescence (red) and the CDs fluorescence (green) partially overlapped (yellow) in the lysosomal region. It can be seen that since the lysosomes were acidic organelles, the fluorescence lifetime of the CDs was relatively shorter when they were distributed in this region (Figure 3b,c), and the fluorescence lifetime distribution from the CDs could be well distinguished. The pH value is 4.0–5.5 in the lysosomes and 7.2–7.4 in the cytoplasm [62]. It should be noted that in Figure 3c, the peaks of the CDs’ fluorescence lifetime in the lysosomes and outside the lysosomes were 1.6 ± 0.5 ns and 2.3 ± 0.3 ns, respectively. Correspondingly, in Figure 1a(ii), the fluorescence lifetime values corresponding to the CDs solution of pH = 5.0 and pH = 7.3 were 2.5 ± 0.1 ns and 3.3 ± 0.1 ns. As can be seen from Figure 2, if the cells were in the pH buffer of pH = 5.0 and pH = 7.3, the average fluorescence lifetime values of the CDs were approximately 2.1 ± 0.1 ns and 2.4 ± 0.2 ns. Compared with the three sets of fluorescence lifetime values (see Table 1), the fluorescence lifetime was relatively close when the CDs were in the neutral environment of the cells (in the neutral buffer treated cells or outside the lysosomes of untreated cells). Both the 2.4 ± 0.2 ns and 2.3 ± 0.3 ns values are lower than the fluorescence lifetime in a neutral aqueous solution (3.3 ± 0.1 ns). When the CDs were in cells in an acidic environment, their fluorescence lifetime (2.1 ± 0.1 ns) was also slightly lower than that of the aqueous solutions at the same pH (2.5 ± 0.1 ns). This may have been caused by the complex micro-environment in the cells. In addition to the pH value, the cells were also rich in various coenzymes, amino acids, proteins, etc., which may have affected the fluorescence properties of the CDs. This will be discussed in detail in the following sections.

As seen in Figure 3d and Supplemental Video S1, the fluorescence lifetime distribution of the CDs in HeLa living cells could be observed from a three-dimensional perspective, where the low fluorescence lifetime corresponded to a region of low pH within the cells. The fluorescence lifetime of the CDs was not uniform in the cells, which further confirms that CDs could respond to complex microenvironments in the cells.

### 3.3. Fluorescence Lifetime of the CDs in the Nucleus

CDs are mainly distributed in the cytoplasm in living cells, but a small amount can enter the nucleus. For example, the fluorescence intensity in nuclei was about 8% of the whole cells in Figure 3. Since HeLa cell is a rapidly dividing type cell line, the cytoplasm to nucleus ratio would be less where the nuclear size would be relatively larger than normal cells. Hence, the probability of any foreign material getting into the nucleus upon disruption of the cell membrane is high. This might result in the CDs distribution in nuclei. Furthermore, as shown in Figure 2a, it can be found that the pH buffer affected cell membrane permeability, as reported in a number of previous studies [63,64,65] allowing more CDs to enter the cells, as well as the nuclei. The change of cell membrane permeability may partially attribute to the pH-sensitive membrane proteins [65]. The fluorescence lifetime of the CDs in the nuclei was longer than that in the cytoplasm.

**Figure 4:**
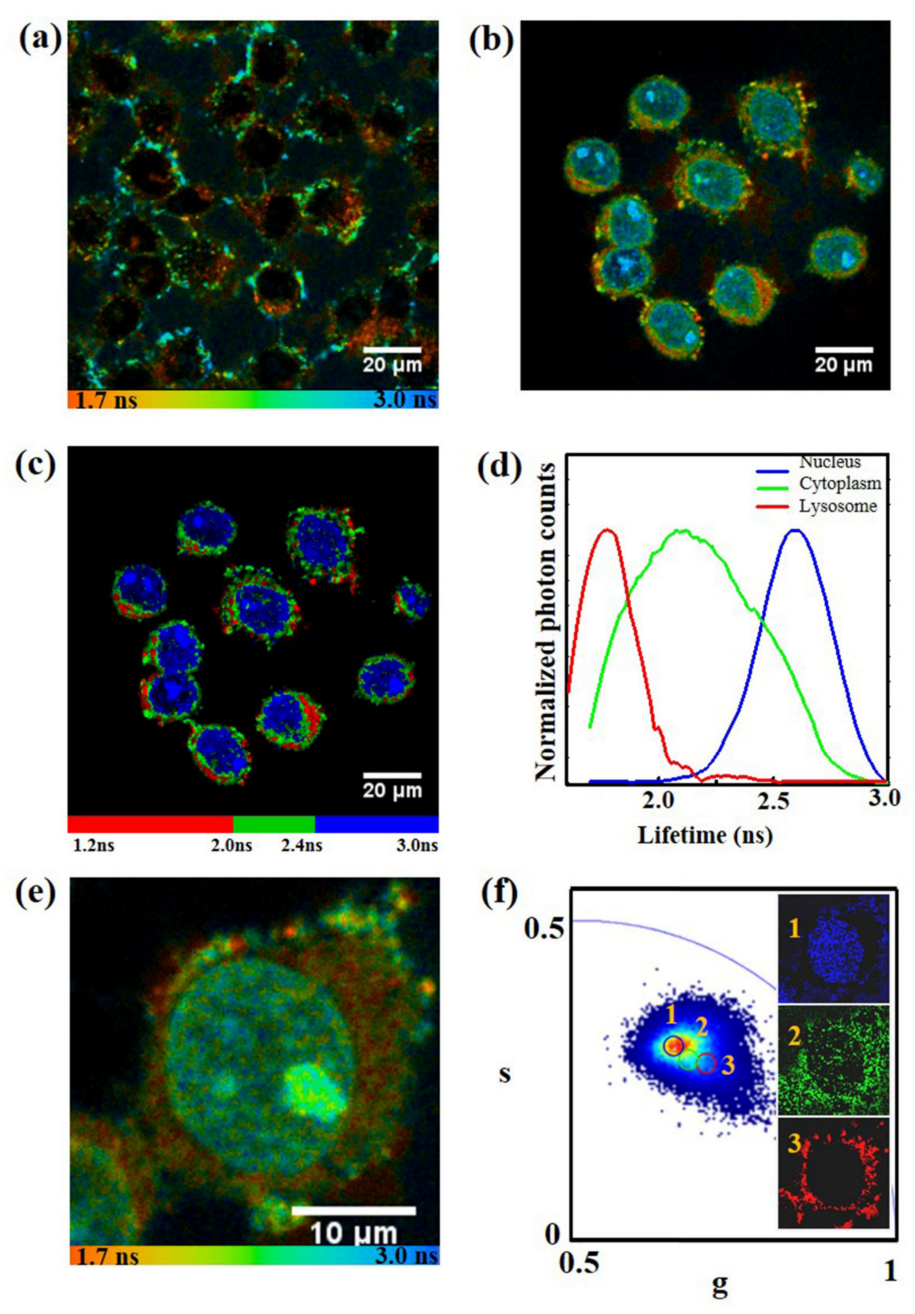
The FLIM images of the CDs in the HeLa cells were compared. (**a**) Cells incubated with CDs for 2 h. (**b**) Cells incubated with CDs for 2 h, and then cultured with digitonin for 3 min for cell permeabilization (scale bar: 20 μm). (**c**) The different fluorescence lifetimes of CDs in the lysosomes, cytoplasm, and nucleus of the cell in discrete colors. (**d**) Statistical curves of the CDs fluorescence lifetime distribution in different regions of the cell (lysosomes, cytoplasm, and nucleus). (**e**) FLIM images of a permeabilized cell and (**f**) the corresponding phasor FLIM distribution, where 1, 2, and 3 correspond to the three regions of the nucleus, cytoplasm, and lysosomes, respectively.

To further observe the fluorescence lifetime of the CDs in the nuclei, digitonin was used to increase the permeability of the cell membrane and nuclear envelope. Figure 4 compares the FLIM images of CDs in living cells, which were not treated with digitonin or treated with digitonin. In Figure 4a, the CDs rarely entered the nucleus when the cell membrane was intact, and the fluorescence lifetime was about 1.6–2.3 ns. When the permeability of the cell membrane and nuclear envelope changed, the CDs could enter the nuclei, and the fluorescence lifetime was longer than that in the cytoplasm, about 2.5–2.8 ns (Figure 4b). Figure 4c,d shows the different fluorescence lifetimes of CDs in the lysosomes (red), cytoplasm (green), and nucleus (blue) of the cell in discrete colors.

Figure 4e presents the FLIM image of CDs in a HeLa cell treated with digitonin, and Figure 4f shows the corresponding phasor image analysis. It can be seen from the Figure 4f that there are three regions representing different distributions, acidic lysosomes, neutral cytoplasm, and the nucleus. There are three circles in Figure 4f corresponding to red, green and blue, respectively, which correspond to the three images below. It is noticed that most of carbon-based dots or semiconductor quantum dots can barely enter the nucleus [34,36,66]. However, the CDs in this work can cross the nuclear envelope, which provides an approach for monitoring the microenvironment in the nucleus.

### 3.4. Effects of Coenzymes, Amino Acids and Proteins on the Fluorescence of the CDs

The cells had a complex microenvironment, including a large number of coenzymes, amino acids, and proteins. We selected a representative coenzyme NADH, two amino acids, and two proteins in order to study their effects on the fluorescence of the CDs. Among them, NADH is an important coenzyme molecule involved in energy metabolism [67]. The acidic amino acids and basic amino acids were represented by glutamic acid and arginine, respectively [68]. The isoelectric points (pI) of histone and bovine serum albumin (BSA) are approximately 10 and 4.7–4.9, respectively. In the pH = 7.0 solution, the histone is positively charged and the BSA is negatively charged. The histone is active in the nucleus of the eukaryotic cells and it participates in cell cycle regulation.

**Figure 5:**
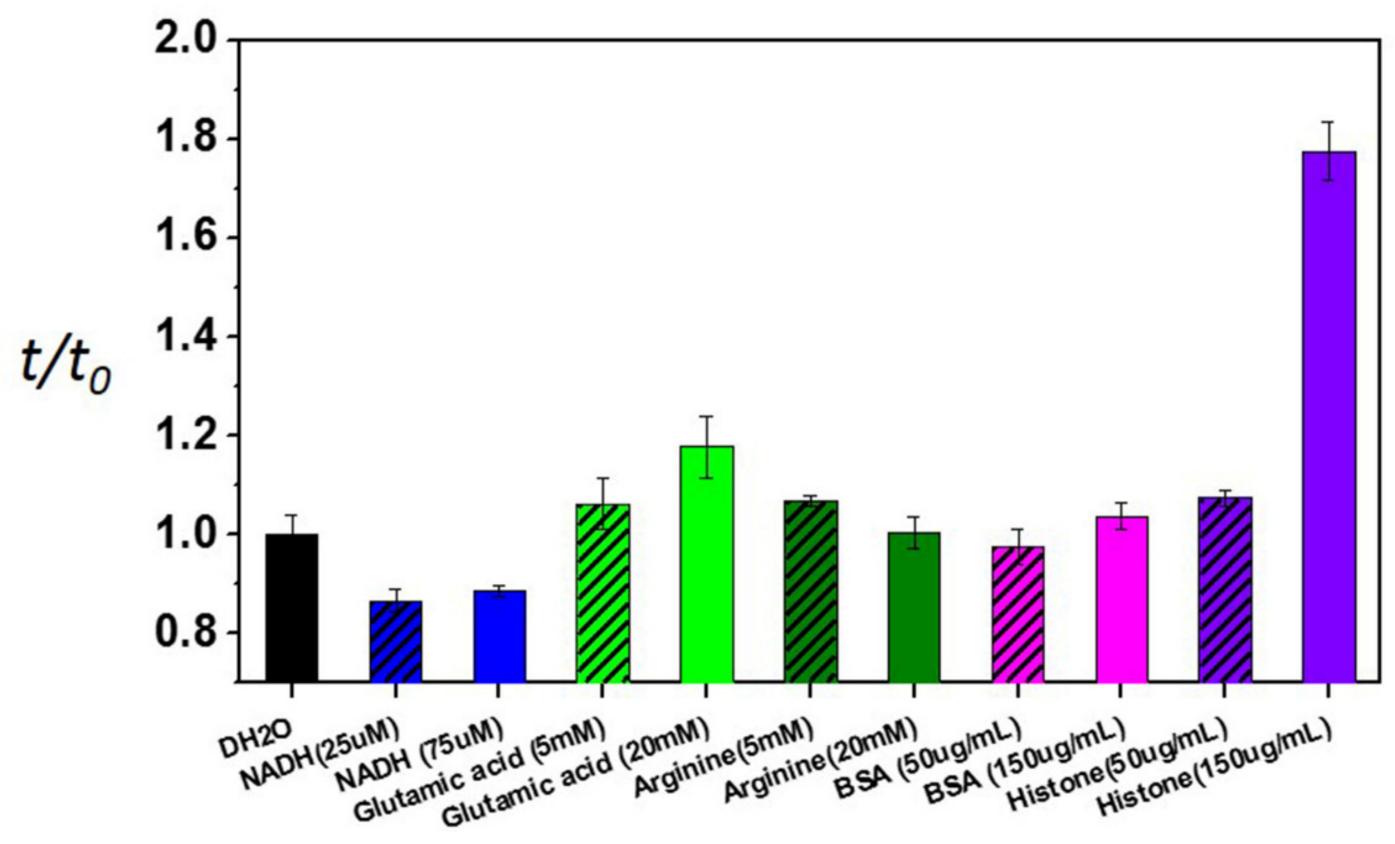
The effects of different concentrations of coenzyme NADH, amino acids, proteins on the fluorescence lifetime of the CDs (*t*_0_ and *t* are the CD fluorescence lifetime in the DH_2_O and other solutions, respectively). Coenzyme NADH reduced the fluorescence lifetime of the CDs. Amino acid Arg and bovine serum albumin (BSA) did not affect the fluorescence lifetime of the CDs. Glutamic acid slightly increased the fluorescence lifetime of the CDs, and 150 μg mL^−1^ histone greatly increased the fluorescence lifetime of the CDs.

As shown in Figure 5, the effect of different substances on the fluorescence lifetime of the CDs solution was compared at pH = 7.0. The coenzyme NADH of 25–75 μM caused a decrease in the CDs fluorescence lifetime of about 12–14%, which might partly explain why the fluorescence lifetime of the CDs in the aqueous solution was slightly longer than that in cells at the same pH. Two amino acids, glutamate and arginine, as well as two proteins, histone and BSA, increased the fluorescence lifetime of the CDs. The effect of histone was the greatest among them. When the histone concentration was 150 μg mL^−1^, the fluorescence lifetime of the CDs increased by 78%. Therefore, it could be considered that the histone in the nucleus contributed greatly to the increase of the fluorescence lifetime of the CDs. When the CDs entered the nucleus and reacted with histone, a higher fluorescence lifetime of the CDs in the nucleus could be observed, as shown in Figure 4. The interaction between CDs and proteins, amino acids or coenzymes may be affected by various factors, including the isoelectric point, the molecular weight, and Zeta potential [69]. In this regard, histone on CDs may be highly selective among them. CDs might be wrapped in the molecules, thereby inhibiting the non-radiative transitions or affecting the type of electronic transitions, then lead to the change of fluorescence lifetime.

## 4. Conclusions

This work demonstrated the pH-sensitive CDs for intracellular pH sensing with FLIM for the first time. The different groups on the CDs surface that changed with the microenvironment led to changes of the fluorescence lifetime. Thus, the fluorescence lifetime of the CDs solutions with different pH values (pH = 2.6–8.6) increased linearly with the increase of pH. The fluorescence decay time also increased against the pH values of the buffer from 3.2 to 8.3 in the pH buffer-treated cells. FLIM imaging and phasor FLIM analysis were applied to observe the CDs in the lysosomes, cytoplasm, and nucleus of living cells. It was shown that the CDs in the lysosomes had a short lifetime of 1.6 ns. It was also shown that the CDs could enter the nuclei, and the fluorescence lifetime was much longer than that in the cytoplasm. In addition, the effects of coenzymes, amino acids, and proteins on the fluorescence lifetime of the CDs were examined. It was found that histone in the nucleus contributed greatly to the increase of the fluorescence lifetime of the CDs. This study provides a useful method of combining pH-sensitive CDs with FLIM technology for intracellular pH sensing in complex biological environments.

## Figures and Tables

**Table 1:** Comparison of fluorescence lifetimes of the CDs under different conditions.

		Fluorescence Lifetimes (ns)		Fluorescence Lifetimes (ns)
Solutions	Ph = 5.0	2.5 ± 0.1	pH = 7.3	3.3 ± 0.1
pH buffer-treated cells	pH = 5.0 (buffer)	2.1 ± 0.1	pH = 7.3 (buffer)	2.4 ± 0.2
Untreated cells	in lysosomes	1.6 ± 0.5	outside lysosomes	2.3 ± 0.3

## Supplementary Materials

The following are available online at https://www.mdpi.com/2079-4991/10/4/604/s1. Figure S1 The cytotoxicity of CDs with the incubation concentrations of 20–50 μg mL^−1^ on HeLa cells. Figure S2 (a) Absorbance and (b) fluorescence spectra of the CDs aqueous solution (0.05 mg mL^−1^). Figure S3 A typical FLIM image of CDs-treated cells (a) and its analysis. (b) The example fitting curves of one pixel in image (a) by single, double, or triple-exponential fitting. (c) The fluorescence average lifetime (t_m_) distribution curve of image (a), in which the peak of the lifetime distribution curve was 2.25 ns. Figure S4 The comparison of (a) autofluorescence in control cells and (b) CDs fluorescence in CDs-treated HeLa cells. (c) The statistical analysis of (a) and (b). Figure S5 (a) Confocal fluorescence images of Oregon Green-labeled HeLa cells in different pH buffers, scale bar: 20 μm. (b) Plot of intensity versus varying pH in pH buffer treated HeLa cells. Table S1 A typical multi-exponential fitting result of fluorescence lifetime data from one pixel in Figure S2. Video S1 Three-dimensional FLIM imaging for CDs-treated living HeLa cells.

## References

[1] Han J., Burgess K. (2009). Fluorescent indicators for intracellular pH. Chem. Rev..

[2] Flinck M., Kramer S.H., Pedersen S.F. (2018). Roles of pH in control of cell proliferation. Acta Physiol..

[3] Lan A., Lagadic-Gossmann D., Lemaire C., Brenner C., Jan G. (2007). Acidic extracellular pH shifts colorectal cancer cell death from apoptosis to necrosis upon exposure to propionate and acetate, major end-products of the human probiotic propionibacteria. Apoptosis.

[4] Di Sario A., Bendia E., Omenetti A., De Minicis S., Marzioni M., Kleemann H., Candelaresi C., Saccomanno S., Alpini G., Benedetti A. (2007). Selective inhibition of ion transport mechanisms regulating intracellular pH reduces proliferation and induces apoptosis in cholangiocarcinoma cells. Dig. Liver Dis..

[5] Hiromu S., Guangshuai L., Yoshiko H., Yoshie M., Junko K., Makoto S., Miyuki K. (2013). Increases in intracellular pH facilitate endocytosis and decrease availability of voltage-gated proton channels in osteoclasts and microglia. J. Physiol..

[6] Parks S.K., Johanna C., Jacques P. (2015). pH control mechanisms of tumor survival and growth. J. Cell. Physiol..

[7] Webb B.A., Michael C., Jacobson M.P., Barber D.L. (2011). Dysregulated pH: A perfect storm for cancer progression. Nat. Rev. Cancer.

[8] Curtain C.C., Ali F.E., Smith D.G., Bush A.I., Masters C.L., Barnham K.J. (2003). Metal ions, pH, and cholesterol regulate the interactions of Alzheimer’s disease amyloid-beta peptide with membrane lipid. J. Biol. Chem..

[9] Bao B., Yang Z., Liu Y., Xu Y., Gu B., Chen J., Su P., Tong L., Wang L. (2019). Two-photon semiconducting polymer nanoparticles as a new platform for imaging of intracellular pH variation. Biosens. Bioelectron..

[10] Zhu H., Fan J., Xu Q., Li H., Wang J., Gao P., Peng X. (2012). Imaging of lysosomal pH changes with a fluorescent sensor containing a novel lysosome-locating group. Chem. Commun..

[11] Zhang J., Li J., Chen B., Kan J., Jiang T., Zhang W., Yue J., Zhou J. (2019). An off-on fluorescent probe for real-time sensing the fluctuations of intracellular pH values in biological processes. Dyes Pigment..

[12] Burgstaller S., Bischof H., Gensch T., Stryeck S., Gottschalk B., Ramadani-Muja J., Eroglu E., Rost R., Balfanz S., Baumann A. (2019). pH-Lemon, a fluorescent protein-based pH reporter for acidic compartments. ACS Sens..

[13] Koktysh D.S. (2020). Ratiometric pH sensor using luminescent CuInS2/ZnS quantum dots and fluorescein. Mater. Res. Bull..

[14] Shangguan J., He D., He X., Wang K., Xu F., Liu J., Tang J., Yang X., Huang J. (2016). Label-Free Carbon-Dots-Based Ratiometric Fluorescence pH Nanoprobes for Intracellular pH Sensing. Anal. Chem..

[15] Jiang M., Gu X., Lam J.W., Zhang Y., Kwok R.T., Wong K.S., Tang B.Z. (2017). Two-photon AIE bio-probe with large Stokes shift for specific imaging of lipid droplets. Chem. Sci..

[16] Chen N., He Y., Su Y., Li X., Huang Q., Wang H., Zhang X., Tai R., Fan C. (2012). The cytotoxicity of cadmium-based quantum dots. Biomaterials.

[17] Nareoja T., Deguchi T., Christ S., Peltomaa R., Prabhakar N., Fazeli E., Perala N., Rosenholm J.M., Arppe R., Soukka T. (2017). Ratiometric sensing and imaging of intracellular pH using polyethylenimine-coated photon upconversion nanoprobes. Anal. Chem..

[18] Chen Y., Lian H., Wei Y., He X., Chen Y., Wang B., Zeng Q., Lin J. (2018). Concentration-induced multi-colored emissions in carbon dots: Origination from triple fluorescent centers. Nanoscale.

[19] Songnan Q., Xiaoyun W., Qipeng L., Xingyuan L., Lijun W. (2012). A biocompatible fluorescent ink based on water-soluble luminescent carbon nanodots. Angew. Chem..

[20] Patidar R., Rebary B., Sanghani D.A., Bhadu G.R., Paul P. (2017). Fluorescent carbon nanoparticles obtained from charcoal via green methods and their application for sensing Fe3+ in an aqueous medium. Luminescence.

[21] Pirsaheb M., Mohammadi S., Salimi A., Payandeh M. (2019). Functionalized fluorescent carbon nanostructures for targeted imaging of cancer cells: A review. Microchim. Acta.

[22] Chandra A., Singh N. (2017). Cell Microenvironment pH Sensing in 3D Microgels Using Fluorescent Carbon Dots. ACS Biomater. Sci. Eng..

[23] Li Y., Zheng X., Zhang X., Liu S., Pei Q., Zheng M., Xie Z. (2017). Porphyrin-Based Carbon Dots for Photodynamic Therapy of Hepatoma. Adv. Healthc. Mater..

[24] Shi H., Wei J., Li Q., Xue C., Meng X. (2014). Fluorescent Carbon Dots for Bioimaging and Biosensing Applications. J. Biomed. Nanotechnol..

[25] Hailong L., Yingwei Z., Lei W., Jingqi T., Xuping S. (2010). Nucleic acid detection using carbon nanoparticles as a fluorescent sensing platform. Chem. Commun..

[26] Peng Z., Miyanji E.H., Zhou Y., Pardo J., Hettiarachchi S.D., Li S., Blackwelder P.L., Skromne I., Leblanc R.M. (2017). Carbon dots: Promising biomaterials for bone-specific imaging and drug delivery. Nanoscale.

[27] Bacakova L., Pajorova J., Tomkova M., Matejka R., Broz A., Stepanovska J., Prazak S., Skogberg A., Siljander S., Kallio P. (2020). Applications of Nanocellulose/Nanocarbon Composites: Focus on Biotechnology and Medicine. Nanomaterials.

[28] Zheng M., Li Y., Liu S., Wang W., Xie Z., Jing X. (2016). One-Pot To Synthesize Multifunctional Carbon Dots for Near Infrared Fluorescence Imaging and Photothermal Cancer Therapy. Acs Appl. Mater. Interfaces.

[29] Zhang T., Dong S., Zhao F., Deng M., Fu Y., Lü C. (2019). Tricolor emissive carbon dots for ultra-wide range pH test papers and bioimaging. Sens. Actuators B.

[30] Kong B., Zhu A., Ding C., Zhao X., Li B., Tian Y. (2012). Carbon Dot-Based Inorganic-Organic Nanosystem for Two-Photon Imaging and Biosensing of pH Variation in Living Cells and Tissues. Adv. Mater..

[31] Wu Z.L., Gao M.X., Wang T.T., Wan X.Y., Zheng L.L., Huang C.Z. (2014). A general quantitative pH sensor developed with dicyandiamide N-doped high quantum yield graphene quantum dots. Nanoscale.

[32] Wang Q., Yang H., Zhang Q., Ge H., Zhang S., Wang Z., Ji X. (2019). Strong acid-assisted preparation of green-emissive carbon dots for fluorometric imaging of pH variation in living cells. Microchim. Acta.

[33] Yao C., Xu Y., Xia Z. (2018). A carbon dot-encapsulated UiO-type metal organic framework as a multifunctional fluorescent sensor for temperature, metal ion and pH detection. J. Mater. Chem. C.

[34] Zhang M., Su R., Zhong J., Fei L., Cai W., Guan Q., Li W., Li N., Chen Y., Cai L. (2019). Red/orange dual-emissive carbon dots for pH sensing and cell imaging. Nano Res..

[35] Liu W., Li C., Sun X., Wei P., Wang J. (2017). Carbon-dot-based ratiometric fluorescent pH sensor for the detections of very weak acids assisted by auxiliary reagents that contribute to the release of protons. Sens. Actuators B.

[36] Chattopadhyay A., Pal A., Ahmad K., Dutta D. (2019). Boron Doped Carbon Dots with Unusually High Photoluminescence Quantum Yield for Ratiometric Intracellular pH Sensing. ChemPhysChem.

[37] Barati A., Shamsipur M., Abdollahi H. (2016). Carbon dots with strong excitation-dependent fluorescence changes towards pH. Application as nanosensors for a broad range of pH. Anal. Chim. Acta.

[38] Sun Y., Wang X., Wang C., Tong D., Wu Q., Jiang K., Jiang Y., Wang C., Yang M. (2018). Red emitting and highly stable carbon dots with dual response to pH values and ferric ions. Microchim. Acta.

[39] Zhang C., Cui Y., Song L., Liu X., Hu Z. (2016). Microwave assisted one-pot synthesis of graphene quantum dots as highly sensitive fluorescent probes for detection of iron ions and pH value. Talanta.

[40] Yuan F., Ding L., Li Y., Li X., Fan L., Zhou S., Fang D., Yang S. (2015). Multicolor fluorescent graphene quantum dots colorimetrically responsive to all-pH and a wide temperature range. Nanoscale.

[41] Orte A., Alvarez-Pez J.M., Ruedas-Rama M.J. (2013). Fluorescence Lifetime Imaging Microscopy for the Detection of Intracellular pH with Quantum Dot Nanosensors. ACS Nano.

[42] Zheng C., An X., Gong J. (2015). Novel pH sensitive N-doped carbon dots with both long fluorescence lifetime and high quantum yield. RSC Adv..

[43] Hille C., Berg M., Bressel L., Munzke D., Primus P., Lohmannsroben H.G., Dosche C. (2008). Time-domain fluorescence lifetime imaging for intracellular pH sensing in living tissues. Anal. Bioanal. Chem..

[44] Battisti A., Digman M.A., Gratton E., Storti B., Beltram F., Bizzarri R. (2012). Intracellular pH measurements made simple by fluorescent protein probes and the phasor approach to fluorescence lifetime imaging. Chem. Commun..

[45] Poea-Guyon S., Pasquier H., Merola F., Morel N., Erard M. (2013). The enhanced cyan fluorescent protein: A sensitive pH sensor for fluorescence lifetime imaging. Anal. Bioanal. Chem..

[46] Qu S.N., Zhou D., Li D., Ji W.Y., Jing P.T., Han D., Liu L., Zeng H.B., Shen D.Z. (2016). Toward Efficient Orange Emissive Carbon Nanodots through Conjugated sp(2)-Domain Controlling and Surface Charges Engineering. Adv. Mater..

[47] Zeng Q., Shao D., He X., Ren Z., Ji W., Shan C., Qu S., Li J., Chen L., Li Q. (2016). Carbon dots as a trackable drug delivery carrier for localized cancer therapy in vivo. J. Mater. Chem. B.

[48] Li D., Jing P., Sun L., An Y., Shan X., Lu X., Zhou D., Han D., Shen D., Zhai Y. (2018). Near-infrared excitation/emission and multiphoton-induced fluorescence of carbon dots. Adv. Mater..

[49] Orij R., Urbanus M.L., Vizeacoumar F.J., Giaever G., Boone C., Nislow C., Brul S., Smits G.J. (2012). Genome-wide analysis of intracellular pH reveals quantitative control of cell division rate by pH(c) in Saccharomyces cerevisiae. Genome Biol..

[50] Tominaga H., Ishiyama M., Ohseto F., Sasamoto K., Hamamoto T., Suzuki K., Watanabe M. (1999). A water-soluble tetrazolium salt useful for colorimetric cell viability assay. Anal. Commun..

[51] Stringari C., Nourse J.L., Flanagan L.A., Gratton E. (2012). Phasor fluorescence lifetime microscopy of free and protein-bound NADH reveals neural stem cell differentiation potential. PLoS ONE.

[52] Yang M., Li B., Zhong K., Lu Y. (2018). Photoluminescence properties of N-doped carbon dots prepared in different solvents and applications in pH sensing. J. Mater. Sci..

[53] Ehtesabi H., Hallaji Z., Nobar S.N., Bagheri Z. (2020). Carbon dots with pH-responsive fluorescence: A review on synthesis and cell biological applications. Microchim. Acta.

[54] Hu Y., Yang J., Tian J., Jia L., Yu J.-S. (2014). Waste frying oil as a precursor for one-step synthesis of sulfur-doped carbon dots with pH-sensitive photoluminescence. Carbon.

[55] Mondal T.K., Saha S.K. (2019). Facile Approach To Synthesize Nitrogen-and Oxygen-Rich Carbon Quantum Dots for pH Sensor, Fluorescent Indicator, and Invisible Ink Applications. ACS Sustain. Chem. Eng..

[56] Mondal T.K., Mondal S., Ghorai U.K., Saha S.K. (2019). White light emitting lanthanide based carbon quantum dots as toxic Cr (VI) and pH sensor. J. Colloid Interface Sci..

[57] Nideep T., Ramya M., Sony U., Kailasnath M. (2019). MSA capped CdTe quantum dots for pH sensing application. Mater. Res. Express.

[58] Sharma V., Tiwari P., Mobin S.M. (2017). Sustainable carbon-dots: Recent advances in green carbon dots for sensing and bioimaging. J. Mater. Chem. B.

[59] Chao S.-C., Wu G.-J., Huang S.-F., Dai N.-T., Huang H.-K., Chou M.-F., Tsai Y.-T., Lee S.-P., Loh S.-H. (2018). Functional and molecular mechanism of intracellular pH regulation in human inducible pluripotent stem cells. World J. Stem Cells.

[60] Persi E., Duran-Frigola M., Damaghi M., Roush W.R., Aloy P., Cleveland J.L., Gillies R.J., Ruppin E. (2018). Systems analysis of intracellular pH vulnerabilities for cancer therapy. Nat. Commun..

[61] Gerbeau P., Amodeo G., Henzler T., Santoni V., Ripoche P., Maurel C. (2002). The water permeability of Arabidopsis plasma membrane is regulated by divalent cations and pH. Plant J..

[62] Martens T.F., Remaut K., Demeester J., De Smedt S.C., Braeckmans K. (2014). Intracellular delivery of nanomaterials: How to catch endosomal escape in the act. Nano Today.

[63] Peiró-Salvador T., Ces O., Templer R.H., Seddon A.M. (2009). Buffers may adversely affect model lipid membranes: A cautionary tale. Biochemistry.

[64] Bouvrais H.l.n., Duelund L., Ipsen J.H. (2014). Buffers affect the bending rigidity of model lipid membranes. Langmuir.

[65] Carroll J.A., Garon C.F., Schwan T.G. (1999). Effects of environmental pH on membrane proteins in Borrelia burgdorferi. Infect. Immun..

[66] Jiao Y., Gong X., Han H., Gao Y., Lu W., Liu Y., Xian M., Shuang S., Dong C. (2018). Facile synthesis of orange fluorescence carbon dots with excitation independent emission for pH sensing and cellular imaging. Anal. Chim. Acta.

[67] Ying W. (2008). NAD+/NADH and NADP+/NADPH in cellular functions and cell death: Regulation and biological consequences. Antioxid. Redox Signal..

[68] Pettiwala A.M., Singh P.K. (2018). A molecular rotor based ratiometric sensor for basic amino acids. Spectrochim. Acta Part A.

[69] Zhang J., Zheng M., Xie Z. (2016). Co-assembled hybrids of proteins and carbon dots for intracellular protein delivery. J. Mater. Chem. B.

