# Supplementary material for: Carbon Dots for Intracellular pH Sensing with Fluorescence Lifetime Imaging Microscopy
Source: Nanomaterials (Basel). 2020 Mar 25;10(4):604. doi: 10.3390/nano10040604 (PMC7221822; doi:10.3390/nano10040604)
Supplement: Supplementary file 1 [file nanomaterials-10-00604-s001.zip › SM/Carbon dots FLIM-SI-revised.docx]

Article

Carbon Dots for Intracellular pH Sensing with Fluorescence Lifetime Imaging Microscopy

Maojia Huang ^1^, Xinyue Liang ^1^, Zixiao Zhang ^1^, Jing Wang ^2^, Yiyan Fei ^1^, Jiong Ma ^1,3,4,^*, Songnan Qu ^5,^* and Lan Mi ^1,^*

^1^ Department of Optical Science and Engineering, Shanghai Engineering Research Center of Ultra-precision Optical Manufacturing, Green Photoelectron Platform, Fudan University, Shanghai 200433, China; [17210720002@fudan.edu.cn](mailto:17210720002@fudan.edu.cn) (M.H.); [18210720003@fudan.edu.cn](mailto:18210720003@fudan.edu.cn) (X.L.); [16307130097@fudan.edu.cn](mailto:16307130097@fudan.edu.cn) (Z.Z.); [fyy@fudan.edu.cn](mailto:fyy@fudan.edu.cn) (Y.F.); [jiongma@fudan.edu.cn](mailto:jiongma@fudan.edu.cn) (J.M.); [lanmi@fudan.edu.cn](mailto:lanmi@fudan.edu.cn) (L.M.)

^2^ State Key Laboratory of High Field Laser Physics, Shanghai Institute of Optics and Fine Mechanics, Chinese Academy of Sciences, Shanghai 201800, China; wangjing@siom.ac.cn

^3^ Institute of Biomedical Engineering and Technology, Academy for Engineer and Technology, Fudan University, Shanghai 200433, China.

^4^ The Multiscale Research Institute of Complex Systems (MRICS), School of Life Sciences, Fudan University, Shanghai 200433, China.

^5^ Joint Key Laboratory of the Ministry of Education, Institute of Applied Physics and Materials Engineering, University of Macau, Avenida da Universidade, Taipa, Macau 999078, China; [songnanqu@um.edu.mo](mailto:songnanqu@um.edu.mo).

***** Correspondence: [lanmi@fudan.edu.cn](mailto:lanmi@fudan.edu.cn); Tel.: +86-21-6564-2092 (L.M.); [songnanqu@um.edu.mo](mailto:songnanqu@um.edu.mo); Tel.: +853-8822-9096 (S.Q.); [jiongma@fudan.edu.cn](mailto:jiongma@fudan.edu.cn); Tel.: +86-21-6564-2134 (J.M.)

Received: 9 February 2020; Accepted: 24 March 2020; Published: date

Supplementary materials:


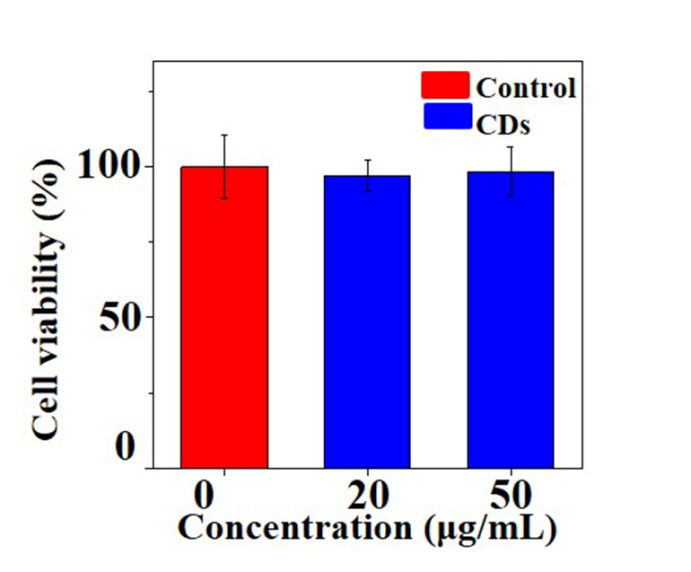


**Figure. S1** The cytotoxicity of CDs with the incubation concentrations of 20-50 μg mL^-1^ on HeLa cells.

**
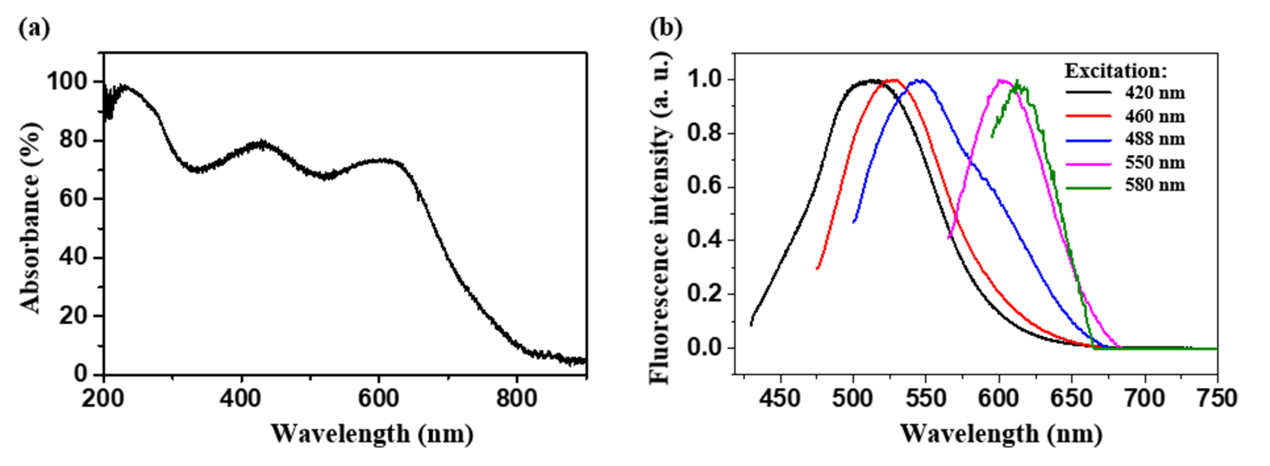
Figure. S2** (a) Absorbance and (b) fluorescence spectra of the CDs aqueous solution (0.05mg mL^-1^).

Fig. S3(b) and Table S1 showed that the triple-exponential fitting was better than single- or double-exponential decay, where $t_{m}=\sum_{i=1}^{N} t_{i}a_{i}$. As listed in Table S1, t_2_ of the double-exponential fitting is between t_2_ and t_3_ of the triple-exponential fitting. The ratio of the latter two components in the triple-exponential fitting (a2 and a3) is relatively large, and cannot be ignore. In addition, χ^2^ is closer to 1 when using triple-exponential fitting. Therefore, triple-exponential fitting is more reflective of the true information of the different components than the double exponential fitting. Thus, the three exponential fitting for the fluorescence lifetime of CDs is used in this paper.


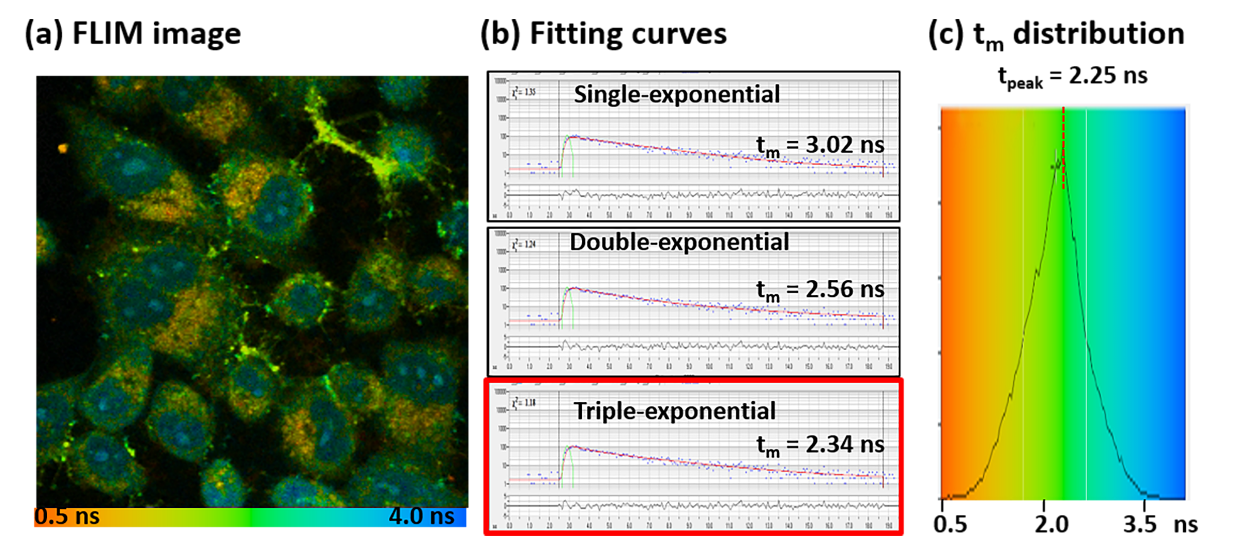


**Figure. S3** A typical FLIM image of CDs-treated cells (a) and its analysis. (b) The example fitting curves of one pixel in image (a) by single, double, or triple-exponential fitting. (c) The fluorescence average lifetime (t_m_) distribution curve of image (a), in which the peak of the lifetime distribution curve was 2.25 ns.

**Table S1** A typical multi-exponential fitting result of fluorescence lifetime data from one pixel in Fig. S2

|  | Single-exponential fitting | Double-exponential fitting | Triple-exponential fitting |
| --- | --- | --- | --- |
| t_m_ (ns) | 3.02 | 2.56 | 2.34 |
| t_1_ (ns) | 3.02 | 1.75 | 0.86 |
| a_1_ | 100% | 76.40% | 45.40% |
| t_2_ (ns) |  | 5.18 | 3.47 |
| a_2_ |  | 23.60% | 12.70% |
| t_3_ (ns) |  |  | 3.59 |
| a_3_ |  |  | 41.90% |
| ^^ | 1.35 | 1.24 | 1.18 |


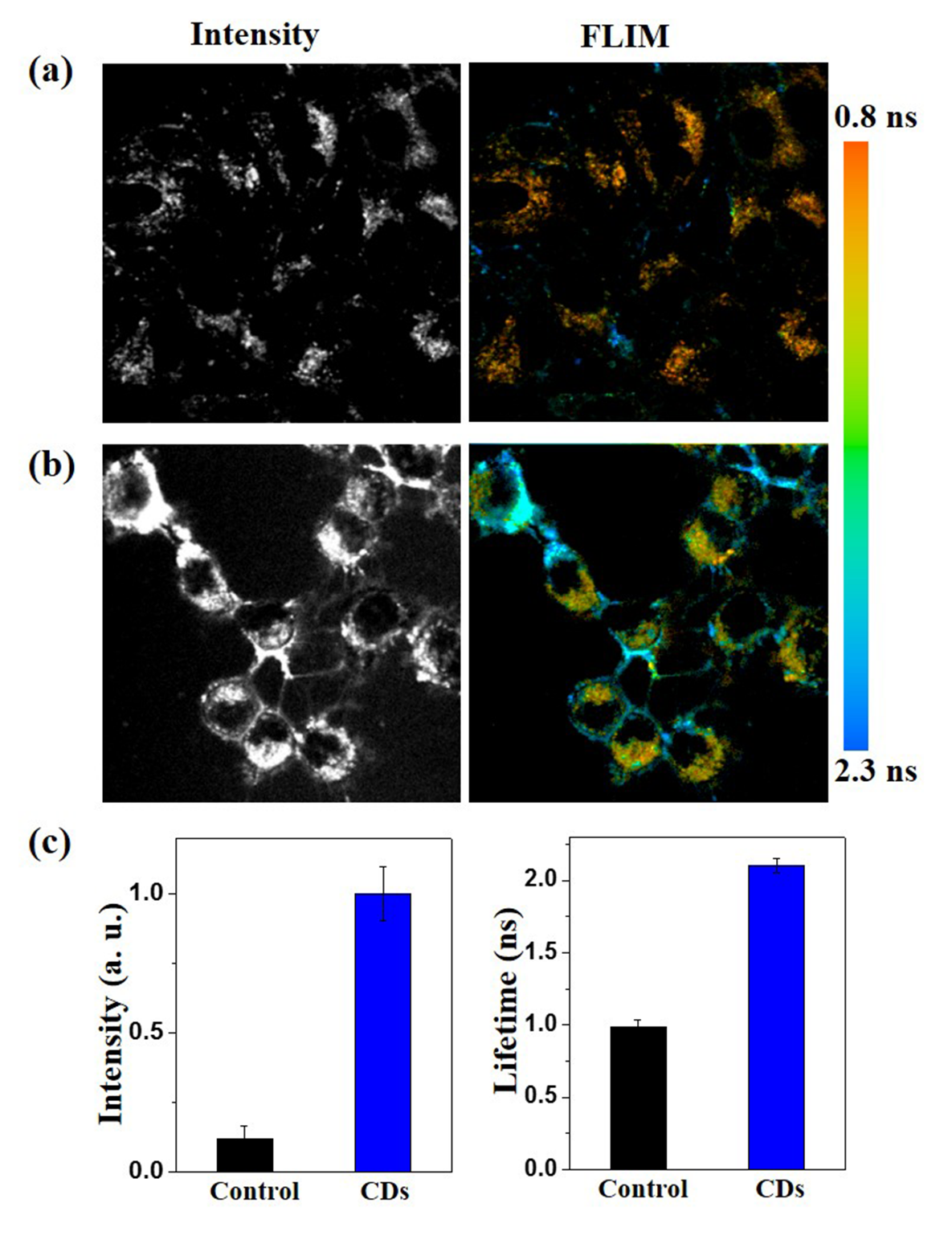


**Figure. S4** The comparison of (a) autofluorescence in control cells and (b) CDs fluorescence in CDs-treated HeLa cells. (c) The statistical analysis of (a) and (b).


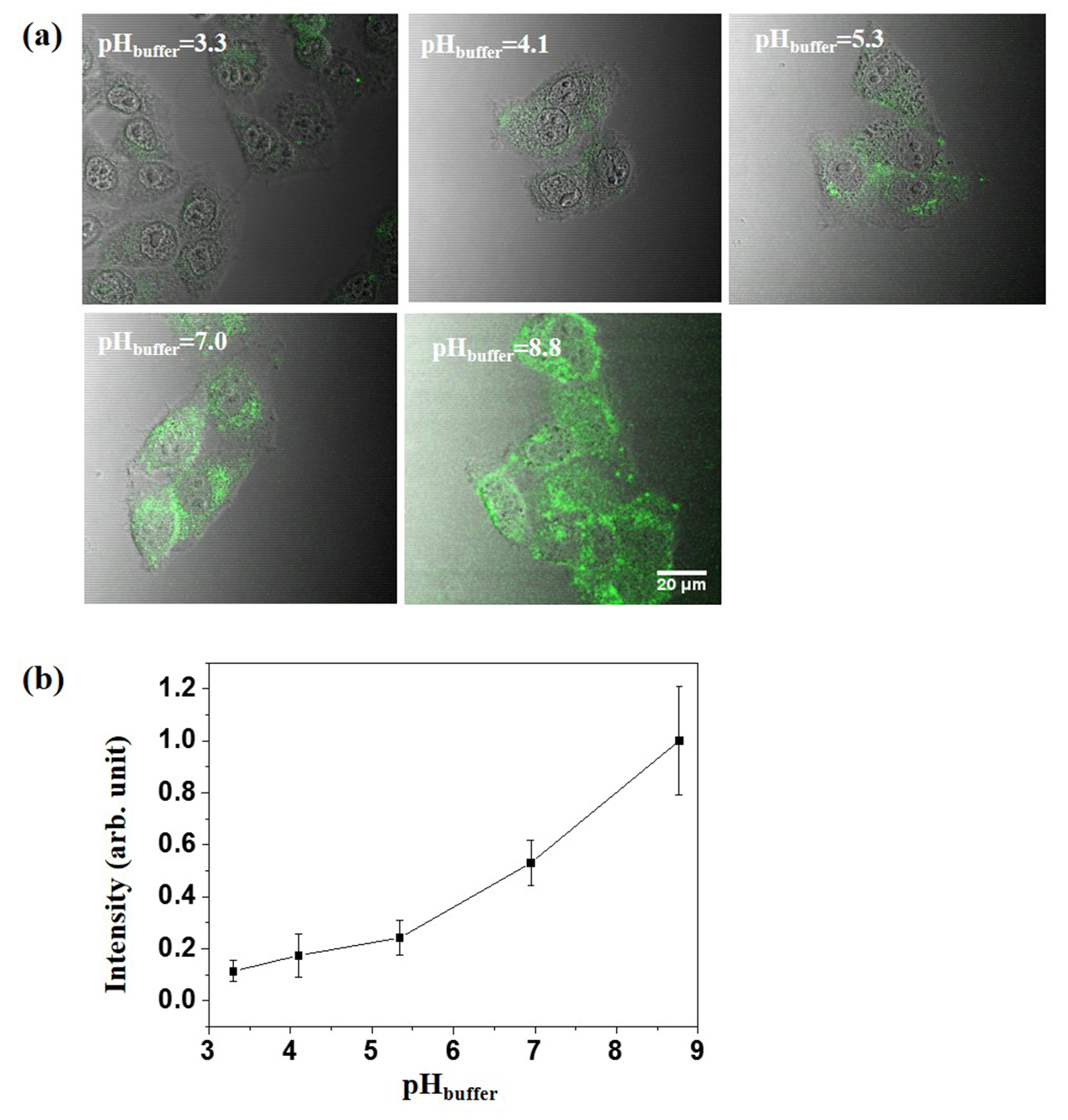


**Figure. S5** (a) Confocal fluorescence images of Oregon Green -labeled HeLa cells in different pH buffers, scale bar: 20 μm. (b) Plot of intensity versus varying pH in pH buffer treated HeLa cells.

**Video S1** Three-dimensional FLIM imaging for CDs-treated living HeLa cells.
